# Hepatitis C Virus Clearance Cascade — United States, 2013–2022

**DOI:** 10.15585/mmwr.mm7226a3

**Published:** 2023-06-30

**Authors:** Carolyn Wester, Ademola Osinubi, Harvey W. Kaufman, Hasan Symum, William A. Meyer, Xiaohua Huang, William W. Thompson

**Affiliations:** ^1^Division of Viral Hepatitis, National Center for HIV, Viral Hepatitis, STD, and TB Prevention, CDC; ^2^Quest Diagnostics, Secaucus, New Jersey; ^3^Office of the Director, National Center for HIV, Viral Hepatitis, STD, and TB Prevention, CDC.

Approximately 2.4 million adults were estimated to have hepatitis C virus (HCV) infection in the United States during 2013–2016 (*1*). Untreated, hepatitis C can lead to advanced liver disease, liver cancer, and death (*2*). The Viral Hepatitis National Strategic Plan for the United States calls for ≥80% of persons with hepatitis C to achieve viral clearance by 2030 (*3*). Characterizing the steps that follow a person’s progression from testing to viral clearance and subsequent infection (clearance cascade) is critical for monitoring progress toward national elimination goals. Following CDC guidance (*4*), a simplified national laboratory results-based HCV five-step clearance cascade was developed using longitudinal data from a large national commercial laboratory throughout the decade since highly effective hepatitis C treatments became available. During January 1, 2013–December 31, 2021, a total of 1,719,493 persons were identified as ever having been infected with HCV. During January 1, 2013–December 31, 2022, 88% of those ever infected were classified as having received viral testing; among those who received viral testing, 69% were classified as having initial infection; among those with initial infection, 34% were classified as cured or cleared (treatment-induced or spontaneous); and among those persons, 7% were categorized as having persistent infection or reinfection. Among the 1.0 million persons with evidence of initial infection, approximately one third had evidence of viral clearance (cured or cleared). This simplified national HCV clearance cascade identifies substantial gaps in cure nearly a decade since highly effective direct-acting antiviral (DAA) agents became available and will facilitate the process of monitoring progress toward national elimination goals. It is essential that increased access to diagnosis, treatment, and prevention services for persons with hepatitis C be addressed to prevent progression of disease and ongoing transmission and achieve national hepatitis C elimination goals.

An 8–12 week short-course of well-tolerated, oral-only treatment with DAA agents is recommended for nearly all persons with HCV infection (*5*) and results in a cure in ≥95% of cases (*6*). A national program to eliminate hepatitis C in the United States was proposed earlier this year (*7*) to provide an opportunity to accelerate national efforts toward eliminating hepatitis C. The Viral Hepatitis National Strategic Plan for the United States calls for ≥80% of persons with hepatitis C to achieve viral clearance by 2030 (*1*). Characterizing the HCV clearance cascade is critical for monitoring progress toward national elimination goals, identifying gaps in care and program effectiveness, and prioritizing public health resource allocations. Developing a comprehensive national hepatitis C care cascade is challenging, because no single data source sufficiently describes all steps of the cascade. Previous HCV care cascades have required using data from a variety of sources (e.g., household surveys, cohort studies, laboratory testing, and pharmacy claims) to inform distinct steps in the cascade (*8*). In response to these challenges, CDC developed guidance for generating a simplified, laboratory results–based HCV clearance cascade (*4*). Following this methodology and using data from a large national commercial laboratory, this report presents a national HCV clearance cascade during the DAA era (January 1, 2013–December 31, 2022).

Data were analyzed from patients living in all 50 states and the District of Columbia who received hepatitis C testing by Quest Diagnostics. Quest Diagnostics programming was applied to de-identify and de-duplicate data. Tests included HCV antibody (anti-HCV), HCV RNA nucleic acid (quantitative or qualitative), and HCV genotype. The HCV clearance cascade characterized persons according to five steps, 1) ever infected, defined as any receipt of a positive HCV test result (i.e., any reactive anti-HCV or detectable HCV RNA or genotype) during January 1, 2013–December 31, 2021 (index period); 2) viral testing, defined as evidence of ≥1 HCV RNA test performed during January 1, 2013–December 31, 2022 (the follow-up period) for a person characterized as having ever been infected; 3) initial infection, defined as evidence of a detectable HCV RNA during the follow-up period in any person with viral testing; 4) cured or cleared, defined (among persons with an initial infection) as evidence of subsequent undetectable HCV RNA during the follow-up period (approximately one third of persons with acute infection will self-clear initial HCV infection without treatment); and 5) persistent infection or reinfection, defined as evidence of subsequent detectable HCV RNA in any person categorized as cured or cleared, during the follow-up period (*4*).

Frequencies of persons at each cascade step were calculated. Conditional proportions for each step were calculated using the number of persons identified who met the definition for being at a particular step divided by the number that met the definition from the previous step, following the methods in the CDC guidance document (*4*).

Persons in each of the HCV clearance cascade steps were analyzed by age group, sex, and payor type. Age group was categorized as 0–19, 20–39, 40–59, and ≥60 years. Payor type was categorized as Medicare, Medicaid, commercial, other (client or self-pay), and unspecified (no payor type provided). This activity was reviewed by CDC and conducted consistent with applicable federal law and CDC policy.*

A total of 1,719,493 persons were identified as ever having been infected. (Figure 1). For subsequent steps, 1,520,592 (88%) of those ever infected were categorized as having had viral testing; 1,042,082 (69%) of those with viral testing were categorized as having an initial infection; 356,807 (34%) of those with initial infection as cured or cleared; and 23,518 (7%) of those categorized as cured or cleared as having persistent infection or reinfection.

**FIGURE 1:**
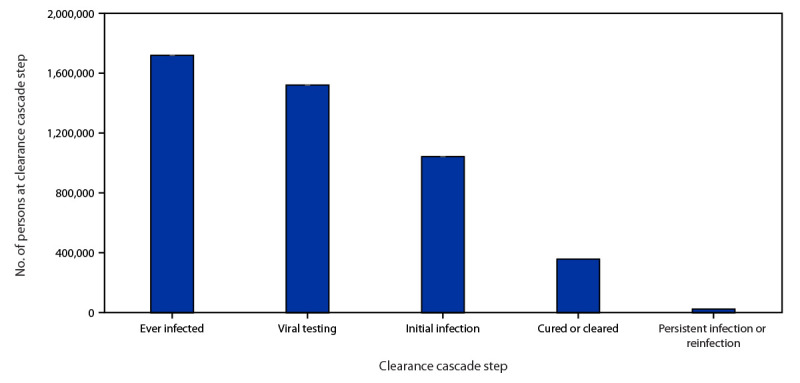
Hepatitis C virus clearance cascade using national commercial laboratory data — United States, 2013–2022 **Source**: Quest Diagnostics (January 1, 2013–December 31, 2022).

Among those ever infected, 29%, 43%, and 27% were persons aged 20–39 years, 40–59 years, and ≥60 years, respectively; 60% were male. Among the 1,719,493 persons ever infected, 862,905 (50%) were covered by commercial health insurance, followed by 386,755 (23%) by other payor, 186,464 (11%) by Medicaid, 151,217 (9%) by unspecified payor, and 132,152 (8%) by Medicare (Table).

**TABLE:** Hepatitis C virus clearance cascade, by selected demographics — United States, 2013–2022

Characteristic	No. of persons at clearance cascade step				
	Ever infected*	Viral testing^†^ (%)^§^	Initial infection^†^ (%)^§^	Cured or cleared^†^ (%)^§^	Persistent infection or reinfection^†^ (%)^§^
**Age group, yrs** ^¶^					
0–19	18,035	15,370 (85.2)	6,274 (40.8)	1,581 (25.2)	107 (6.8)
20–39	490,190	434,922 (88.7)	304,022 (69.9)	72,362 (23.8)	6,644 (9.2)
40–59	738,534	650,353 (88.1)	458,284 (70.5)	167,835 (36.6)	10,813 (6.4)
≥60	472,319	419,640 (88.8)	273,329 (65.1)	114,995 (42.1)	5,953 (5.2)
**Sex** ^¶^					
Male	1,031,819	910,407 (88.2)	663,711 (72.9)	226,208 (34.1)	17,622 (7.8)
Female	682,383	606,891 (88.9)	376,033 (62.0)	130,147 (34.6)	5,868 (4.5)
**Payor type**					
Medicare	132,152	119,693 (90.6)	76,719 (64.1)	34,356 (44.8)	1,993 (5.8)
Medicaid	186,464	164,324 (88.1)	112,654 (68.6)	34,817 (30.9)	2,009 (5.8)
Commercial	862,905	783,199 (90.8)	496,429 (63.4)	196,789 (39.6)	15,125 (7.7)
Other (self-pay or client bill)	386,755	333,335 (86.2)	258,425 (77.5)	58,548 (22.7)	2,306 (3.9)
Unspecified	151,217	120,041 (79.4)	97,855 (81.5)	32,297 (33.0)	2,085 (6.5)

* The ever infected category was assessed during the baseline period of January 1, 2013–December 31, 2021.

^†^ The viral testing, initial infection, cured or cleared, and persistent infection or reinfection categories were assessed during the follow-up period of January 1, 2013–December 31, 2022.

^§^ Conditional proportion based on immediately preceding column.

^¶^ Age and sex weren’t reported for 415 and 5,291 persons, respectively, in the ever infected category.

The prevalence of viral testing ranged from 79% (unspecified payor) to 91% (commercial and Medicare payors). Initial infection was lowest among those aged 0–19 years (41%); payor type ranged from 63% for those with commercial insurance to 82% for those with unspecified payor type. The prevalence of being cured or cleared was lowest among persons aged 20–29 years (24%), and highest among those aged ≥60 years (42%). By payor type, cured or cleared prevalences ranged from 23% for other to 45% for Medicare.

Hepatitis C viral clearance increased with age when stratified by payor type among those with initial infection (Figure 2). The lowest proportion of cured or cleared, across all age groups, was among those with other payor (range = 16%–29%), followed by unspecified (20%–41%) and Medicaid (23%–38%), and then by commercial (29%–49%) and Medicare (33%–46%) payors. The highest proportion of cured or cleared among all age groups and payors was 49% for commercially insured persons aged ≥60 years. Persistent infection or reinfection was highest among persons aged 20–39 years (9%).

**FIGURE 2:**
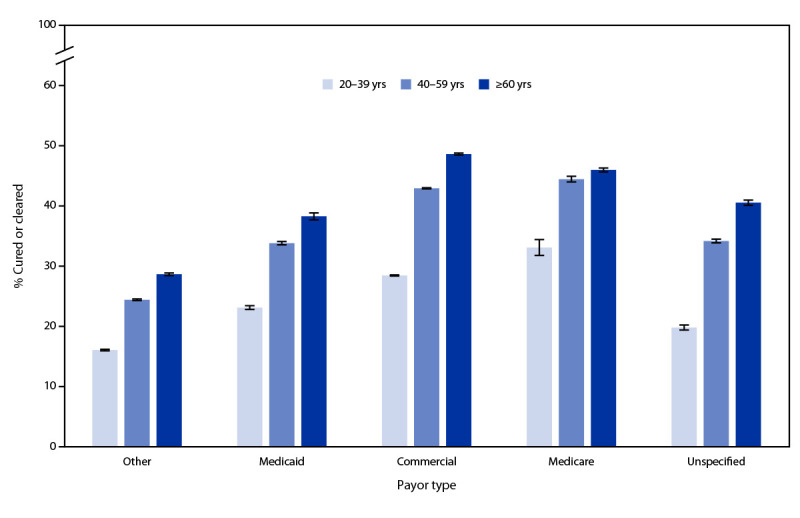
Proportion* of hepatitis C virus–infected persons with evidence of viral clearance, by age group^†^ and payor type^§^ — United States, 2013–2022^¶^ **Source**: Quest Diagnostics (January 1, 2013–December 31, 2022). * With 95% CIs indicated by error bars. ^†^ Persons aged 0–19 years were not included because of small sample sizes. ^§^ Other payor includes client or self-pay, and unspecified includes persons with no payor type provided in the record. ^¶^ Includes all persons with initial infection during January 1, 2013–December 31, 2022.

## Discussion

Using U.S. longitudinal commercial laboratory data, this report presents an HCV clearance cascade with data for approximately 1.7 million persons with evidence of a history of HCV infection during the DAA era. Analysis revealed that 88% of persons with evidence of a history of HCV infection received viral RNA testing, and among the 1.0 million persons with evidence of initial infection, approximately one third had evidence of viral clearance (cured or cleared); 7% of those with viral clearance had evidence of subsequent viremia (persistent infection or reinfection). These findings reveal substantial missed opportunities to diagnose, treat, and prevent hepatitis C in the United States.

Among the approximately 1.0 million persons in this analysis with initial infection, only 34% had laboratory evidence of viral clearance. Persons with other, unspecified, or Medicaid payor type had lower viral clearance (23%, 33%, and 31%, respectively) than persons with Medicare and commercial payors (40% and 45%, respectively). These observations are consistent with recently published hepatitis C treatment coverage data among insured adults (*9*) and highlight the large gap between current viral clearance and the nation’s goal of ≥80% viral clearance among persons with diagnosed hepatitis C by 2030 (*3*).

Overall, viral clearance was lowest among persons aged 20–39 years (24%). Within this age group, those with other, unspecified or Medicaid payor type had lower viral clearance prevalences (16%, 20%, and 23%, respectively) than did persons with Medicare and commercial payors (33% and 29%, respectively). Similarly, persistent infection or reinfection was highest among persons aged 20–39 years (9%). These findings highlight the disproportionate need for increased access to hepatitis C treatment and prevention services among younger adults.

 The application of commercial laboratory data to this simplified, standard, laboratory result–based HCV clearance cascade fills a substantial data gap nationally, including assessments of complete diagnosis, viral clearance, and subsequent viremia. This analysis, using one large commercial laboratory can be easily updated, and along with the large sample size, provides the precision needed to follow trends over time. Identifying and quantifying progress and gaps in the HCV clearance cascade will help guide the implementation of hepatitis C diagnosis, treatment, and prevention activities in support of national hepatitis C elimination goals.

The findings in this report are subject to at least six limitations. First, the results were based on a population of persons who received a positive test result for HCV and do not represent all persons with HCV infection. Second, data from a single laboratory are not necessarily nationally representative. Nevertheless, during 2013–2022, approximately 1.0 million persons in this analysis were identified with initial infection, consistent with approximately 42% of the estimated 2.4 million persons to have hepatitis C in the United States (*1*). Third, the follow-up period is not uniform, which might contribute to variations in rates along steps in the cascade. Fourth, the cascade does not capture persons who did not receive a subsequent HCV RNA test after initial infection, those being cured or cleared, or those having persistent infection or reinfection, and therefore, likely underestimates the number and percentage of persons within these steps of the cascade. Fifth, persons who received HCV laboratory testing from both Quest Diagnostics and other laboratories would not have the other laboratory results represented in these estimates, which could lead to different estimates reported in each step. Finally, using other care cascade models might be preferable when prevalence and comprehensive diagnosis and treatment data are available.

 Increased access to diagnosis, treatment, and prevention services for persons with or at risk for acquiring hepatitis C needs to be addressed to prevent progression of disease and ongoing transmission, and to achieve national hepatitis C elimination goals. Overcoming these barriers requires implementation of universal hepatitis C screening recommendations including HCV RNA testing for all persons with reactive HCV antibody results, provision of treatment for all persons regardless of payor, and prevention services for persons at risk for acquiring new HCV infection.

**Summary**

**What is already known about this topic?**

The Viral Hepatitis National Strategic Plan for the United States calls for ≥80% of persons with hepatitis C to achieve viral clearance by 2030. Assessing progress toward elimination goals requires monitoring hepatitis C virus (HCV) clearance.

**What is added by this report?**

An analysis of the HCV clearance cascade using 2013–2022 national HCV testing data found that the prevalence of viral clearance among persons with diagnosed hepatitis C was only 34% overall and was even lower (16%) among persons aged 20–39 years with other payor (client or self-pay) insurance.

**What are the implications for public health practice?**

Increased access to diagnosis, treatment, and prevention services for persons with hepatitis C would prevent progression of disease and ongoing transmission and achieve national hepatitis C elimination goals.

## Footnotes

* 45 C.F.R. part 46, 21 C.F.R. part 56; 42 U.S.C. Sect. 241(d); 5 U.S.C. Sect. 552a; 44 U.S.C. Sect. 3501 et seq.
